# Capillary Blood Docosahexaenoic Acid Levels Predict Electrocardiographic Markers in a Sample Population of Premenopausal Women

**DOI:** 10.3390/jcm13195957

**Published:** 2024-10-07

**Authors:** Breno P. Casagrande, George Sherrard, Mike S. Fowler, Débora Estadella, Allain A. Bueno

**Affiliations:** 1 College of Health, Life and Environmental Sciences, University of Worcester, Henwick Grove, Worcester WR2 6AJ, UK; breno.casagrande@unifesp.br (B.P.C.); georgiehome@yahoo.co.uk (G.S.); 2 Biosciences Department, Institute of Health and Society, Federal University of São Paulo, Santos 1015-020, SP, Brazil; estadella@unifesp.br; 3 Department of Biosciences, Swansea University, Singleton Park, Swansea SA2 8PP, UK; m.s.fowler@swansea.ac.uk

**Keywords:** N-3 polyunsaturated fatty acid, docosahexaenoic acid, blood fatty acid, QRS, mediation analysis

## Abstract

**Introduction**: The relationship between blood N-3 polyunsaturated fatty acid (PUFA) levels and cardiovascular health is known, but direct evidence that N-3 PUFA levels influence electrocardiographic (ECG) parameters is non-existent. In the study described herein, we investigated the relationship between anthropometric biomarkers and capillary blood PUFAs with ECG outputs in a sample population of healthy pre-menopausal women. **Method**: Twenty-three consenting females were recruited, with the study power analysis sufficiently demonstrated. Food intake, anthropometric and cardiovascular parameters were obtained. Capillary blood was collected for fatty acid chromatographic analysis. **Results**: Body mass index, haematocrit, heart rate (HR), mean arterial pressure (MAP) and ECG readings all fell within healthy ranges. Principal component analysis-mediated correlations were carried out controlling for combined Components 1 (age, body fat % and waist-to-hip ratio) and 2 (height, HR and MAP) as control variables. Docosahexaenoic acid (DHA) unequivocally decreased the QRS area under the curve (AUC-QRS) regardless of the impact of control variables, with each unit increase in DHA corresponding to a 2.3-unit decrease in AUC-QRS. Mediation analysis revealed a significant overall effect of DHA on AUC-QRS, with the impact of DHA on R wave amplitude accounting for 77% of the total observed effect. **Discussion**: Our new findings revealed an inverse relationship between AUC-QRS with capillary blood DHA, suggesting that the association between ventricular mass and its QRS depolarising voltage is mediated by DHA. Our findings bridge a knowledge gap on the relationship between ventricular mass and ventricular efficiency. Further research will confirm whether the relationship identified in our study also exists in diseased patients.

## 1. Introduction

Ischaemic heart diseases (IHDs) are the leading cause of global mortality [1] and are increasing in frequency regionally in the USA and the UK [2,3]. The growing burden on public health systems is compounded by IHD risk factors linked to other overlapping health risks, namely obesity, smoking, sedentariness, hypertension, type II diabetes, and dyslipidaemia [4,5,6,7,8,9,10].

Dietary components influence the aetiology of IHDs, including high intake of sodium, total fat, trans fats, saturated fats, high glycaemic index foods and high N-6 polyunsaturated fatty acid (PUFA) intake [11,12,13,14,15,16,17] concomitant to low fibre and low N-3 PUFA intake [18,19]. Adherence to healthier diets is known to exert cardioprotective benefits [20,21,22]. Recommendations on saturated fatty acid (SFA) intake for the prevention of heart disease remain contentious [23]. Nonetheless, in their Scientific Opinion on Dietary Reference Values for fats, the European Food Safety Authority recommends the intake of SFA for adults to be as low as possible [24]. As important components of the Mediterranean diet, monounsaturated fatty acids (MUFAs) present some important anti-inflammatory and lipid-lowering properties when associated with other cardioprotective dietary compounds [25,26]. N-3 and N-6 PUFAs are essential fatty acids with fundamental roles in cell membrane composition and function, paracrine and endocrine signalling, and inflammation and its resolution [27,28,29].

Diets poor in N-3 are associated with elevated cardiometabolic and vascular risk, whereas N-3-adequate diets provide protection to the cardiovascular system [30,31,32,33]. N-3 metabolites possess anti-inflammatory properties, which are known to improve vascular endothelium function [34,35]. N-3 PUFAs are thought to interact in an advantageous manner with the sodium and potassium ion channel membrane proteins that are key to action potential propagation during the cardiac cycle [36]. Importantly, n-3 PUFAs have been shown to reduce baseline heart rate and increase resting heart rate variability in dogs [37], healthy men [38], and myocardial infarction-recovered men [39].

Despite our understanding of the beneficial effects of n-3 PUFAs on overall cardiovascular health, direct evidence that N-3 blood levels have the potential to influence electrocardiographic (ECG) parameters is yet to be identified. We hypothesise that such a potential relationship, where N-3 fatty acids influence ECG parameters, would further explain mechanisms that associate IHD with diet and mortality. Additionally, as electrocardiography is widespread in clinical settings, a further interpretation of ECG outputs would add value to therapeutic and disease-preventative strategies.

Our observational study investigated whether anthropometric biomarkers and capillary blood fatty acid profile were associated with ECG output parameters in a free-living sample population of healthy pre-menopausal women. Our hypothesis remained focused on investigating the relationship between blood N-3 PUFAs and ventricular depolarisation determined via lead II-generated QRS duration, R wave amplitude, and area under the curve (AUC) for the QRS segment.

## 2. Materials and Methods

### 2.1. Sample Size Calculation

Considering the number of predictors and control variables for the main objective of this study, the a priori power analysis suggested a sample size between 19 and 29 participants, achieving a moderate effect size f^2^ = 0.3–0.5 (r^2^ = 0.231–0.333) with 80% power (0.8). Calculations were performed using G*Power (3.1) software.

### 2.2. Ethical Approval and Participants

The University of Worcester Health, Life and Environmental Sciences Research Ethics Panel approved this research (CHLES18190021-R, 17 June 2019). Pre-menopausal women residing in Worcestershire, England, were invited to participate in this study through social media and flyers. All participants understood all risks associated with this research, had all their questions answered to their full satisfaction by the research team, voluntarily agreed to take part in it and signed a consent form. There were no financial incentives of any form. Participants’ personal details and data were handled under the General Data Protection Regulation, implemented by the United Kingdom Data Protection Act 2018. Confidentiality, anonymity, data gathering, processing, storage, protection, and disposal were conducted under the University of Worcester Health Research policy. All information collected from participants were anonymised before analyses, and no participant can be identified in the present report.

Inclusion criteria included the female sex with gender identity matching gender assignment at birth, 18 years of age or older, and able to consent. Non-inclusion criteria included smoking or having smoked in the two years prior to participation in the study, any form of liver, kidney, lung, or heart disease, type 2 diabetes, psychiatric disorders, therapies with blood thinners of any type, beta-blockers or any other medication known to affect heart rhythm and vitamin and fatty acid supplementation.

### 2.3. Procedures

Data collection was conducted in the morning, and participants were instructed to have their usual breakfast at home before attending the University of Worcester laboratory for data collection. Questionnaires were completed, and anthropometric measurements, ECG measurements and blood samples were taken.

Height was assessed in cm with a Leicester Height Measure Mk II stadiometer, body mass was assessed in kg with a Seca 760 mechanical scale (Birmingham, UK) and body mass index (BMI) was calculated as kg/m^2^. Body fat % was estimated using an Omron BF306 handheld body composition monitor (Omron Healthcare Inc., Bannockburn, IL, USA). Waist and hip circumferences were assessed in cm with a tape measure following the World Health Organization’s anthropometrical guidelines [40], and their waist-to-hip ratio (WHR) was calculated.

All participants sat in a padded chair with back support for 5 min before cardiovascular assessment and were asked to remain silent and breathe normally during measurements. Peripheral Oxygen Saturation (SpO_2_) and heart rate (HR) were measured with a fingertip pulse oximeter. With both feet flat on the ground and the legs uncrossed, blood pressure was measured using an Omron automatic blood pressure monitor (Omron Healthcare Europe B.V., Hoofddorp, The Netherlands). Systolic (SBP) and diastolic (DBP) blood pressure were recorded and mean arterial pressure (MAP) was calculated as [DBP + 1/3(SBP − DBP)]. Electrocardiographic readings were obtained in the supine position on a padded stretcher using a Seca CT8000i electrocardiograph (Seca GmbH, Hamburg, Germany). Electrocardiographic readings of a minimum of twenty full cardiac cycles were recorded in manual mode from the standard bipolar limb leads I, II and III.

To control for potential confounding variables related to the ECG, we accounted for age, height, body fat %, HR, MAP and WHR.

### 2.4. Blood Sample Collection

Participants were asked to wash their hands with soap in lukewarm tap water prior to the finger prick to improve peripheral vasodilation. Their chosen fingertip was wiped with 2% chlorhexidine and 70% ethanol skin wipes, and two well-rounded capillary blood samples were collected through a finger prick using sterile automatic lancets. The first blood sample was collected into non-heparinised BRAND^®^ micro haematocrit capillary tubes (Merck, Darmstadt, Germany) and immediately centrifuged for 5 min at 8000 rpm. Haematocrit was calculated as erythrocyte to whole blood volume %. The second blood sample was taken onto sterile Standard Grade 3 Whatman filter paper (Whatman, UK), desiccated in a silica gel-filled vacuumed chamber kept in full darkness for 48 h and stored at −80 °C for later analysis.

### 2.5. Capillary Blood Lipid Transmethylation, Fatty Methyl Ester Extraction and Analysis

Lipid transmethylation, fatty acid methyl ester (FAME) extraction and gas chromatographic analysis were performed as described previously by our group [41]. Briefly, each piece of blood-impregnated desiccated filter paper was placed into glass methylating tubes. Two sets of quality control samples were prepared, one of which contained sterile Whatman filter paper and the other of which contained no paper. Four mL of freshly prepared 15% acetyl chloride in dried methanol solution was added. Each tube was flushed under oxygen-free N2 (OFN), airtight cap secured and placed in an oven at 70 °C for 3 h. After 1 and 2 h, the tubes were vortexed and checked for the evaporation of methylating reagent. Where evaporation had occurred, levels were topped up to the marked line with dried methanol before being returned to the oven. Once removed from the oven and cooled, 4 mL 5% NaCl solution and 2 mL HPLC-grade 40–60 °C petroleum spirit containing 0.01% butylated hydroxytoluene (BHT) were added. The tubes were shaken vigorously and quickly spun for clear phase separation. The petroleum layer was transferred to a clean test tube containing 2 mL of 2% KHCO_3_. The samples were washed twice more with 1 mL each of petroleum, collected into the respective test tube, further dried in anhydrous Na_2_SO_4_, transferred to single-use glass vials, evaporated to complete dryness under OFN, resuspended in 0.5 mL 0.01% BHT heptane and stored at −20 °C until analysis. All procedures were performed under subdued light, all solvents were HPLC-grade solvents obtained from Fisher Scientific UK (Loughborough, UK) and all chemicals were obtained from Sigma-Aldrich (Dorset, UK). The samples were injected into a Shimadzu GC2010 Plus Flame Ionization Detector Gas Chromatograph (Shimadzu Corporation, Kyoto, Japan), coupled to Peak Scientific zero air, N2 and H2 generators (Peak Scientific Instruments, Inchinnan, UK), and fitted with an SGE Analytical Science™ BPX70 GC capillary column (60 m × 0.25 mm × 0.25 µm; Milton Keynes, UK). Authentic FAME standards (Supelco 18919-1 FAME mixture (Merck Life Science, Gillingham, UK), Larodan LA90-1210 FAME mixture (Karolinska Institute, Stockholm, Sweden), as well as Sigma individual FAMEs (Dorset, UK)), consisting of a library of 44 individually identifiable FAMEs, were injected to determine the retention time. Fatty acid peaks were unequivocally identified against the retention time of authentic standards. The AUC for each peak was determined using Shimadzu LabSolutions (version 5.82 SP1) software (Shimadzu Corporation, Kyoto, Japan), with the calculation of area % equivalent to weight %. Equal weight standards were used to regulate the instrument response to be equivalent across all FAMEs, as described previously by our group [41].

### 2.6. Diet Analysis

Standardised food diary templates were provided to the participants, who recorded their food and liquid intake over a consecutive four-day period, covering two weekend days and two weekdays. The food diaries were analysed using Nutritics (Nutritics Research Edition, v5.64, Dublin, Ireland) with outputs giving amounts of each nutritional component reportedly consumed per day. The four daily totals were averaged for each participant, with one final figure for each nutrient.

### 2.7. Electrocardiographic Reading Analysis

ECG printouts covering a minimum of 20 cardiac cycles for each participant were digitally scanned in high resolution and the various measurements were quantified using Adobe Acrobat Pro software (version 2022.003.20282). We were interested in data generated by lead II only, with our rationale based on Einthoven’s law [42]. Likewise, precordial derivations and augmented unipolar limb leads aVR, aVL and aVF were not one of our study aims as we were interested in examining electrophysiological hallmarks of myocardial depolarisation and repolarisation only. We were not interested in the time relationships between different waves of the cardiac cycle, which are useful for the diagnosis of cardiac arrythmias, and therefore, such analyses fell outside the remit of our investigation. For those reasons and based on the healthy population recruited for this study, we focused our attention on examining the electrocardiographic parameters generated by lead II only.

Lead II-generated parameters assessed included the AUC for the QRS complex (AUC-QRS), PR interval, QRS duration, P wave duration, QT duration, R wave amplitude and P wave amplitude. The AUC-QRS was measured from the lowest point of the Q wave to the lowest point of the S wave and recorded in mm^2^. X-axis measurements were converted from mm to milliseconds (ms) based on a 25 mm/s recording velocity. Y-axis measurements were converted from mm to millivolts (mV) based on a 10 mm/mV recording amplitude. Individual readings for each parameter for each participant were averaged.

### 2.8. Statistical Analysis

Data were collected and anonymised before tabulation in Microsoft Excel^®^ (Microsoft 365 version 2210) for Windows. Analysis was conducted using jamovi (version 2.4.6), JASP (version 0.17.3) and R (v. 4.2.1) [43]. The data were screened for outliers using the boxplot method, and residual plots were also examined, with no extreme values or influential outliers detected. Descriptive statistics for participant characteristics were calculated and are presented as the mean and standard deviation or as the median and inter-quartile range (P25, P75), depending on the distribution characteristics.

Principal component analysis (PCA) with varimax rotation was used to reduce dimensionality and simplify the interpretation of the component structure. The varimax rotation was selected to enhance the interpretability of the extracted components.

Partial correlations between ECG parameters and anthropometric, dietary and blood fatty acid parameters were performed, controlling for PCA Components 1 and 2. Multiple linear regressions were employed to further explore relationships between variables, considering PCA Components 1 and 2 as potential confounders. Assumptions of normality and homoskedasticity of residuals were checked for each analysis, and no violations were detected.

Mediation analysis was conducted using Components 1 and 2 as covariates. The results of this analysis, including pathway coefficients, *p*-values and statistical power (1-β), are detailed in Supplementary Table S3.

For correlation, regression and mediation analyses, percentile bootstrap confidence intervals (PBCI) were used as they offer better control over type I errors compared to bias-corrected bootstrap confidence intervals (BCBCIs) [44]. A significance threshold of 5% was used for all statistical tests. Correlation results are reported with values of r, *p* and power (1-β), while regression analyses provide standardised and unstandardised effect estimates, 95% confidence intervals, *p*-values and power (1-β).

## 3. Results

Twenty-three women volunteered to participate in the study in full, and no data are missing. The sample population consisted exclusively of pre-menopausal females with gender assigned at birth matching gender identity in all cases. According to the a priori power analysis, the achieved sample size of 23 recruits demands a minimum power of 0.8, despite being significant (*p* < 0.05). The full dietary analysis, presented as 4-day average daily food consumption, is presented in Supplementary Table S1.

### 3.1. Anthropometric Data

Summarised anthropometric and cardiovascular data are presented in Table 1. The median age was 38 (IQR 35, 39). The median BMI was classified as normal (24.5, IQR 22.15, 27.25). The averaged waist circumference (80 ± 8.5 cm) fell within the 2008 WHO Expert Consultation on Waist Circumference and Waist–Hip Ratio reference values [45]. The averaged body fat content was estimated at 36.29 ± 5.11%. Haematocrit, heart rate, SpO_2_, SBP, DBP and MAP all fell within expected normal ranges.

### 3.2. Electrocardiographic Analysis

All ECG readings for the study participants were individually assessed (AAB) for anomalous readings. During the recruitment phase, one participant presented anomalies in their ECG readings; that person was advised to seek medical advice and was excluded from the study. The lead II-generated electrocardiographic parameters quantified are presented in Table 1. All measurements fell within the expected ranges.

### 3.3. Blood Fatty Acid Profile

The averaged and dispersion values for capillary blood fatty acid profile are summarised in Table 2. The SFA family comprised 35.3 ± 2.8% of the total fatty acids, with palmitic acid being the most abundant (23.3 ± 2.1%). The MUFA family comprised 25.2 ± 3% of the total, with being oleic acid the most abundant (20.4 ± 2.3%). The N-6 PUFA family comprised 37.9 ± 4.2% of the total, with linoleic acid being the most abundant (22.7 ± 3.5%), followed by arachidonic acid (8.8 ±1.5%). The N-3 PUFA family comprised 4.9 ± 0.8% of the total, with docosahexaenoic acid (DHA) being the most abundant (2.9 ± 0.6%). The averaged N-6:N-3 ratio was 6.7 ± 0.9 (Table 2 for summarised results; Supplementary Table S2 for full results).

### 3.4. ECG Partial Correlations

The following analyses were controlled for Components 1 and 2. In Component 1, the raw loadings were age (−0.745), body fat % (0.876) and WHR (0.484). In Component 2, the loadings were height (0.413), heart rate (0.791) and MAP (0.730).

We found relevant correlations between lead II-generated ECG phases and blood fatty acids, as well as dietary elements (Table 3 and Table 4). Table 3 shows the identified correlations for the AUC-QRS, QRS duration and R wave amplitude. In summary, AUC-QRS was directly correlated with blood N-6:N-3 ratio and inversely correlated with dietary total fat, energy intake and magnesium intake. QRS duration was directly correlated with dietary total fat and inversely correlated with blood palmitoleic acids. The R wave amplitude was directly correlated with blood N-6:N-3 ratio, dietary total fat and kcal and inversely correlated with blood DHA and total N-3 fatty acids.

Table 4 shows identified the correlations between PR interval, P wave duration and amplitude and QTc (corrected using HR, Bazett formula), with blood fatty acids and dietary components.

### 3.5. Blood DHA and AUC-QRS

The inverse relationship identified between blood DHA and the AUC-QRS and the R wave amplitude were investigated further as it suggests an unexplored link of clinical significance. The partial correlation analyses presented in Table 3 were carried out controlling for PCA-combined Components 1 (age, body fat % and WHR) and 2 (height, HR and MAP). Possible additional confounding variables were tested to exclude the potential effect of other parameters on the identified relationship.

**Figure 1:**
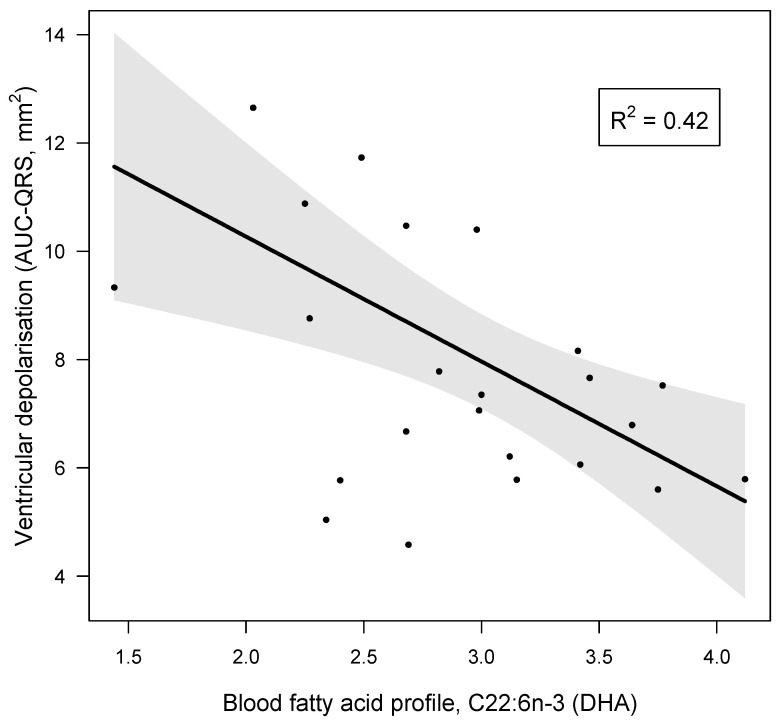
Increasing values of capillary blood docosahexaenoic acid (C22:6n-3, DHA) are associated with a significant reduction in ventricular depolarisation (AUC-QRS), after accounting for variation in other common anthropometric biomarkers. The regression slope and 95% CIs (shaded interval) are based on an additive multiple linear regression model with DHA and the first two axes from a PCA of six common biomarkers as predictor variables, including Factor 1 (age, body fat % and WHR) and Factor 2 (height, HR and MPA). Only DHA showed a significant relationship with AUC-QRS; further statistical model details are provided in Table 5. Figure 1 was created with the visreg package for R [46].

Firstly, we tested as confounders those variables which presented a significant correlation with AUC-QRS (Table 3). Subsequently, we tested as confounders the dietary variables that were correlated with blood DHA, namely trans-fat (g), total kcal, carbohydrate (g), fibre (g), sugars (g) and vitamin D (ug). None of these variables significantly interfered with DHA’s effect on AUC-QRS. More so, none of the other assessed dietary components known to influence the aetiology of IHD [11,12,13,14,15,16,17,18] influenced the DHA and AUC-QRS relationship. Therefore, DHA independently modulated AUC-QRS in our sample population. The final multiple linear regression model is presented in Figure 1 and Table 5.

### 3.6. Mediation Analysis

**Figure 2:**
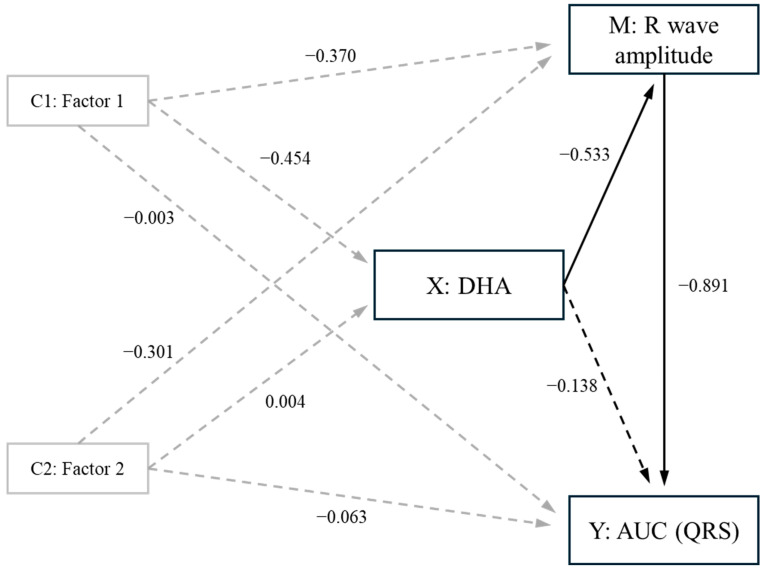
Graphical representation of the mediation model for the effect of DHA on AUC-QRS. C1 and C2: confounder variables, factor 1 and factor 2; (X) predictor: DHA; (Y) outcome: AUC-QRS; (M) mediator: R wave amplitude. Numbers on arrows between model components are the standardised beta values (effects). Dashed lines show non-significant paths; continuous lines show significant paths. Grey lines show control variable effects; black lines show main model effects.

As the R wave amplitude showed an inverse correlation with DHA (Table 3) and is directly related to AUC-QRS (r = 0.947, *p* < 0.001, pw > 0.999), we employed mediation analysis (Mediation Models) to examine whether the effects of DHA on AUC-QRS were due to the effect of DHA on R wave amplitude. The mediation analysis revealed that while there was a significant overall effect of DHA on AUC-QRS (std effect = −0.632, *p* = 0.001, pw = 0.935), the impact of DHA on R wave amplitude mediated this effect (std effect = −0.485, *p* = 0.002, pw = 0.860), accounting for 77% of the total effect. Accordingly, the remaining direct effect of DHA on AUC-QRS was not significant (std effect = −0.131, *p* = 0.291, pw = 0.291) (Figure 2, with significant and non-significant paths presented as solid (*p* < 0.05) and dashed (*p* > 0.05) lines; Supplementary Table S3). The mediation analysis was adjusted for Components 1 and 2, with the findings showing that DHA influenced AUC-QRS by reducing the amplitude of the R wave. Notably, since R wave amplitudes are a constituent of AUC-QRS, the mediation model was able to pinpoint the specific component of AUC-QRS on which DHA had an effect (Figure 2).

## 4. Discussion

Herein, we report for the first time an inverse correlation between the electrocardiographic lead II-generated AUC for the QRS segment with capillary blood DHA content, in which higher blood DHA levels were associated with smaller AUC-QRS readings. The R wave amplitude also showed an inverse correlation with DHA and AUC-QRS. In the same way that left ventricular mass is proportional to electrocardiographic QRS voltage [51], and that increased left ventricular mass disproportionate to electrocardiographic QRS voltage is associated with cardiac fibrosis and myocardial amyloid infiltration [52], our rationale is that higher blood DHA levels exert a protective mechanism over the ventricle, affording a more efficient relationship between ventricular mass and its electrocardiographic QRS depolarising voltage.

Clinical explanations for increased R wave amplitude include physical fitness at rest and ventricular hypertrophy, whilst decreased R wave amplitude is associated with myocardial ischaemia, emphysema, recurrent airway obstructions and chronic wasting diseases. None of our participants had a condition that could explain an abnormal decrease in their R wave amplitude; none were smokers for a minimum of 2 years prior to taking part in this study; no participant was under medical care or taking medicines or dietary supplements; all 23 participants were healthy premenopausal females with their sex assigned at birth matching their sex identity. Their median BMI was classified as normal (Table 1), and the averaged waist circumference fell within WHO reference values. Their ECG readings, haematocrit, heart rate, SpO_2_, SBP, DBP and MAP all fell within expected normal ranges (Table 1 and Table 2).

Dietary data were obtained through a 4-day self-reported food diary. Gersovitz et al. [53] described four days to be sufficient for intake data collection as participants’ motivation to continue recording information tends to wane if the period is made any longer. Our population presented an averaged N-6:N-3 intake ratio of 5.55 ± 4.57 (Supplementary Table S1). There are no official recommendations for the N-6:N-3 intake ratio; nonetheless, the ratio observed is arguably acceptable when compared to reported ratios as high as 25:1 in Western diets [54,55].

Direct transmethylation of finger prick capillary blood provides a fatty acid profile that accurately reflects circulating fatty acids determined through whole blood venipuncture sampling analysis [56,57,58]. As the fatty acid profile of blood components is directly associated with the fatty acid profile of the brain, retina and erythrocytes in humans and in rats [59,60], and the DHA content in heart phospholipids is statistically similar to that of the serum in Wistar rats [61], it is perfectly reasonable to suggest that DHA content in human cardiomyocytes is reflected by DHA concentration in the peripheral blood.

The anti- and pro-arrhythmic properties of N-3 fatty acids have been debated, with evidence suggesting that DHA appears to block the depolarising L-type calcium and Nav1.5 sodium channels and the repolarising Kv1.5 and Kv11.1 potassium channels [36]. Supporting evidence is presented by the inverse correlation identified between elevated T-wave alternans assessed via 24 h Holter ECG and the low serum eicosapentaenoic acid (EPA)/arachidonic acid (AA) ratio in a sample population of IHD men aged 66.3 ± 13.2 years [62], with the authors suggesting that a low EPA/AA ratio could be related to cardiac electrical instability. Such a relationship is not entirely clear, but it appears to be dependent on the incorporation of DHA into the actual cardiomyocytes, and it appears that DHA may directly interact with ion channel proteins at the membrane level.

In addition to directly interacting with ion channels, DHA may also exert cardioprotective effects by modulating membrane lipid composition and altering the biophysical properties of cardiomyocyte membranes [63]). Such effects can influence the function of membrane proteins, such as ion channels, possibly modulating cellular excitability and conduction velocity. DHA has been shown to enhance membrane fluidity, which may stabilise electrical activity and reduce the likelihood of arrhythmogenic disturbances [64]. Furthermore, its well-documented anti-inflammatory and anti-oxidative properties may further support cardiac stability by decreasing the number of pro-inflammatory cytokines and regulating oxidative imbalance [65]. Although the proposed pathways provide a reasonable explanation for the inverse relationship observed between DHA levels and QRS duration, further research is needed to elucidate the specific molecular mechanisms involved in such a relationship.

Considering all analysed parameters, DHA had a unique effect in decreasing AUC-QRS by lowering the R wave amplitude. Each unit increase in DHA was associated with a 2.3-unit reduction in AUC-QRS (Table 5, Figure 1), which corresponds to approximately one standard deviation of our participants’ AUC-QRS. Furthermore, the mediation analysis showed that blood DHA depended on its impact in lowering R wave amplitude, which also decreased approximately one standard deviation per unit increase in DHA.

Mediation models, as presented in Figure 2, provide a way to break down observed effects into direct and indirect effects, helping us understand how much of the observed effect is directly caused by one factor and how much is indirectly influenced through another factor acting as a mediator. In simpler terms, mediation models help us to see how a middleman influences the relationship between two other factors. As the R wave amplitude is part of the AUC-QRS, we tested whether the effect of DHA on the former could have been mediated by other components. We found that no other parameters obtainable from our readings met the criteria for the mediation to be performed, i.e., they were not predicted by DHA. Therefore, the mediation analysis showed that the impact of DHA is on the depolarisation of the main mass of the ventricles (R wave) rather than on the initial depolarisation of the interventricular septum (Q wave) or the depolarisation of the more terminal regions (S wave). The R wave is the greatest contributor to the QRS complex, where the largest number of cardiomyocytes are depolarising at one time, and our data show that the amplitude of the R wave had an adjusted predictive power of 87% on the AUC-QRS complex (r = 0.931, r^2^ = 0.867, *p* < 0.001, pw > 0.999), i.e., it represents the great majority of the area under the curve.

In conclusion, our study has identified for the first time an inverse relationship between blood DHA and AUC-QRS obtained from lead II electrocardiographic readouts in a sample population of healthy premenopausal women. Each unit increase in DHA corresponded with a 2.3-unit decrease in AUC-QRS. Mediation analysis showed that the effect of DHA on AUC-QRS was significantly mediated by the impact of the former on R wave amplitude, accounting for 77% of the total effect. Our findings suggest that higher DHA levels may have a protective effect on the ventricle by improving the efficiency of the relationship between ventricular mass and electrocardiographic QRS voltage.

Our findings are limited to premenopausal women recruited from Worcestershire County, England. Further research is needed to determine whether the relationship identified here is unique to our sample population or present in other groups as well. Factors such as dietary patterns, body composition, physical activity levels, fitness, age, preexisting conditions, disease susceptibility and genetic influences should all be considered. Additionally, it is equally important to investigate this newly identified relationship in other populations, including men, elderly populations, IHD-vulnerable individuals and diseased individuals.

## Figures and Tables

**Table 1:** Anthropometric, cardiovascular, and electrocardiographic data for the sample population.

Anthropometric Parameters	Mean (SD) or Median (IQR)	Typical Values and Expected Ranges
Age ^#^	38.00 (IQR 35.00, 39.00)	
Height (cm)	166.73 (SD: 7.03)	
Weight (kg)	71.96 (SD: 14.72)	
Waist circumference (cm)	80.04 (SD: 8.55)	≤80 [46]
Hip circumference (cm)	104.28 (SD: 9.55)	
Waist–hip ratio	0.77 (SD: 0.04)	<0.85 [46]
Body fat %	36.29 (SD: 5.11)	
BMI (kg/m^2^) ^#^	24.50 (IQR 22.15, 27.25)	18.5–24.9
Haematocrit (%)	39.52 (SD: 2.35)	36–46
Heart rate (bpm)	67.91 (SD: 8.15)	60–100
SpO_2_ (%)	98.00 (SD: 0.60)	95–100
SBP (mmHg)	117.52 (SD: 10.68)	90–120
DBP (mmHg) ^#^	73.00 (IQR 70.50, 85.50)	60–80
MAP (mmHg)	90.46 (SD: 8.46)	70–100
Electrocardiographic parameters		
AUC for the QRS complex (mm^2^)	7.74 (SD: 2.23) [47]	
QRS duration (ms) ^#^	84.80 (IQR 73.60, 89.60) [48]	70–104 [48]
R wave amplitude (mV)	1.08 (SD: 0.32) [47]	<2 mV [47]
PR interval (ms)	149.13 (SD: 21.06) [49]	118–212 [49]
P wave duration (ms)	93.30 (SD: 12.80) [48]	<110 [48]
P wave amplitude (mV)	0.14 (SD: 0.03) [47]	<0.25 mV [47]
QT interval (ms)	393.06 (SD: 20.51) [47]	388–450 [47]
QTc ^#^	408 (IQR 395.98, 432.97) [50]	419 (377, 464) [50]

(BMI) body mass index; (bpm) beats per minute; (DBP) diastolic blood pressure; (IQR) inter-quartile range, p25 and p75; (MAP) mean arterial pressure; (SBP) systolic blood pressure; (SD) standard deviation; (AUC) area under the curve. ^#^ indicates a non-gaussian distribution and the use of median and IQR. All values for ECG parameters fell within the expected ranges. [47] Rautaharju et al., 2013; [48] Meek and Morris, 2002; [49] Vepsäläinen et al., 2014; [50] Rijnbeek et al., 2014. n = 23.

**Table 2:** Capillary blood fatty acid profile (% of total fatty acids) determined using GC-FID.

Fatty Acid (% of Total Fatty Acids)	Mean (SD)	Reference Values ^#^
Total SFAs	35.308 (SD: 2.829)	36.8 (SD: 1.5)
Palmitic acid (C16:0)	23.332 (SD: 2.119)	22.7 (SD: 1.9)
Total MUFAs	25.193 (SD: 3.000)	24.3 (SD: 2.5)
Oleic acid (C18:1n-9)	20.483 (SD: 2.374)	20.0 (SD: 2.5)
Total PUFAs	37.890 (SD 4.189)	
Total n-6	32.939 (SD: 3.685)	33.2 (SD: 1.8)
Arachidonic acid (C20:4n-6)	8.769 (SD: 1.501)	8.1 (SD: 1.6) ^§^
Total n-3	4.953 (SD: 0.788)	4.5 (SD: 1.3)
Docosahexaenoic acid (C22:6n-3)	2.909 (SD: 0.642)	2.8 (SD: 1.1)
n-6:n:3 ratio	6.752 (SD: 0.924)	

Full data are presented in Supplementary Table S2. (GC-FID) gas chromatography—flame ionisation detection; (SFA) saturated fatty acid; (MUFA) monounsaturated fatty acid; (PUFA) polyunsaturated fatty acid; (SD) standard deviation. ^§^ indicates *p* < 0.05 (Z-test) compared to the values found by ^#^ Min et al. (2011) who conducted a capillary blood fatty acid analysis on Whatman Standard Grade 3 filter paper, the one used in our study. n = 23.

**Table 3:** Statistically significant partial correlations with the lead II-generated ECG phases that represent ventricular depolarisation.

Parameters	ECG Phase		
	AUC (QRS)	QRS Duration	R Wave Amplitude
Blood fatty acids			
C16:0 (%)		r = −0.495, *p* = 0.031, pw = 0.736	
C16:1n-7 (%)		r = −0.732, *p* = 0.006, pw = 0.998 *	
C22:6n-3 (DHA)	r = −0.668, *p* = 0.007, pw = 0.983 *		r = −0.612, *p* = 0.016, pw = 0.940 *
Total n-3 (%)	r = −0.615, *p* = 0.033, pw = 0.944 *		r = −0.561, *p* = 0.042, pw = 0.869 *
n6:n3 ratio	r = 0.687, *p* = 0.004, pw = 0.990 *		r = 0.671, *p* = 0.006, pw = 0.984 *
Dietary analysis			
Total fat (g)	r = 0.678, *p* = 0.001, pw = 0.990 *	r = 0.527, *p* = 0.008, pw = 0.889 *	r = 0.539, *p* = 0.009, pw = 0.829 *
Saturated fat (g)	r = 0.523, *p* = 0.013, pw = 0.797		
Total Kcal	r = 0.568, *p* = 0.005, pw = 0.881 *		r = 0.567, *p* = 0.004, pw = 0.879 *
Carbohydrates (g)	r = 0.428, *p* = 0.032, pw = 0.578		r = 0.441, *p* = 0.017, pw = 0.609
Fibre (g)	r = 0.511, *p* = 0.012, pw = 0.772		r = 0.491, *p* = 0.012, pw = 0.727
NSP (g)	r = 0.438, *p* = 0.027, pw = 0.602		r = 0.424, *p* = 0.021, pw = 0.568
Sugars (g)			r = 0.444, *p* = 0.043, pw = 0.616
Glucose (g)			r = 0.498, *p* = 0.025, pw = 0.743
Potassium (mg)	r = 0.423, *p* = 0.005, pw = 0.566		r = 0.449, *p* = 0.008, pw = 0.628
Calcium (mg)	r = 0.490, *p* = 0.008, pw = 0.725		r = 0.423, *p* = 0.023, pw = 0.566
Magnesium (mg)	r = 0.570, *p* = 0.002, pw = 0.884 *		r = 0.478, *p* = 0.011, pw = 0.697
Carotene (µg)			r = 0.423, *p* = 0.049, pw = 0.566
Vitamin E (mg)		r = 0.534, *p* = 0.011, pw = 0.819 *	

Partial correlations were carried out with PCA-combined Components 1 (age, body fat % and WHR) and 2 (height, HR and MAP) as adjusted variables. Percentile bootstrap-corrected confidence intervals were used to calculate *p*-values. (pw) Power, 1-β error; (AUC) area under the curve for the QRS complex; (DHA) docosahexaenoic acid; (ECG) electrocardiogram; (HR) heart rate; (MAP) mean arterial pressure; (NSP) non-starch polysaccharides; (PCA) principal component analysis; (WHR) waist-to-hip ratio. * indicates significative correlations with power above the required value (0.8). n = 23.

**Table 4:** Statistically significant correlations with the remaining lead II-generated ECG phases.

Parameters	PR Interval (ms)	P Wave Duration (ms)	P Wave Amplitude (mV)	QTc (Bazzet Formula)
Weight (kg)	r = 0.851, *p* = 0.008, pw > 0.999 *	r = 0.716, *p* = 0.013, pw = 0.997 *		
Blood fatty acids				
C14:0 (%)		r = −0.570, *p* = 0.011, pw = 0.874 *	r = −0.574, *p* = 0.007, pw = 0.890 *	
C18:0 (%)		r = 0.641, *p* = 0.033, pw = 0.962 *	r = 0.752, *p* = 0.001, pw = 0.999 *	
C24:0 (%)			r = 0.466, *p* = 0.008, pw = 0.669	
Total SFAs (%)	r = 0.544, *p* = 0.016, pw = 0.839 *		r = 0.600, *p* = 0.004, pw = 0.926 *	
C18:1n-9 (%)				
Total MUFAs (%)				r = 0.486, *p* = 0.023, pw = 0.716
C18:2n-6t (9t,12c) (%)	r = −0.542, *p* = 0.011, pw = 0.835 *			
C20:3n-3 (%)				r = 0.494, *p* = 0.015, pw = 0.734
Dietary analysis				
SFA (g)				r = −0.409, *p* = 0.033, pw = 0.532
MUFAs (g)				r = −0.491, *p* = 0.004, pw = 0.727
n-6:n-3		r = −0.663, *p* = 0.029, pw = 0.981 *		
Total KCal				r = −0.439, *p* = 0.005, pw = 0.239

Partial correlations were carried out with PCA-combined Factors 1 (age, body fat % and WHR) and 2 (height, HR and MAP) as adjusted variables. Percentile bootstrap-corrected confidence intervals were used to calculate *p*-values; (pw) power, 1-β error; (MUFA) monounsaturated fatty acid; (SFA) saturated fatty acid; (PCA) principal component analysis. * indicates significative correlations with power above the required value (0.8). n = 23.

**Table 5:** Multiple linear regression model of the additive impact of key predictors of ventricular depolarisation (AUC-QRS, mm^2^).

Parameter Estimates (Coefficients)			95% Confidence Intervals						
Names	Estimate	SE	Lower	Upper	β	df	t-Value	*p*-Value	pw
(Intercept)	7.741	0.381	6.961	8.496	0.018	19	20.291	<0.001	
C22:6n-3 (DHA)	−2.304	0.693	−3.410	−0.753	−0.668	19	−3.005	0.007 *	0.983
Component 1	−0.888	0.444	−1.618	0.0719	−0.391	19	−1.741	0.098	0.490
Component 2	−0.654	0.390	−1.621	0.270	−0.312	19	−1.731	0.100	0.321

Omnibus model fit measures: r^2^ = 0.419, F_(3,19)_ = 4.564 and *p* = 0.014. Shapiro–Wilk’s normality test of residuals: W = 0.987, *p* = 0.988. (SE) Standard error. (pw) Power, 1-β error. * indicates significative correlations with power above the required value (0.8). n = 23.

## Data Availability

The data presented in this study are openly available in [66], available at at https://eprints.worc.ac.uk/14186/ (accessed on 9 August 2024), reference number [14186], University of Worcester Research and Publications.

## Supplementary Materials

The following supporting information can be downloaded at: https://www.mdpi.com/article/10.3390/jcm13195957/s1. Table S1: Full dietary analysis, 4-day average consumption for each nutritional component. Table S2: Complementary to Table 2: Peripheral blood fatty acid profile (% of fatty acids) determined by GC-FID. Table S3: Mediation model and Model information.

## References

[1] Wu P., Yu S., Wang J., Zou S., Yao D.S., Xiaochen Y. (2023). Global burden, trends, and inequalities of ischemic heart disease among young adults from 1990 to 2019: A population-based study. Front. Cardiovasc. Med..

[2] Roth G.A., Mensah G.A., Johnson C.O., Addolorato G., Ammirati E., Baddour L.M., Barengo N.C., Beaton A.Z., Benjamin E.J., Benziger C.P. (2020). GBD-NHLBI-JACC Global Burden of Cardiovascular Diseases Writing Group. Global Burden of Cardiovascular Diseases and Risk Factors, 1990–2019: Update from the GBD 2019 Study. J. Am. Coll. Cardiol..

[3] Office for National Statistics Deaths Registered in England and Wales. https://www.ons.gov.uk.

[4] Kenchaiah S., Evans J.C., Levy D., Wilson P.W.F., Benjamin E.J., Larson M.G., Kannel W.B., Vasan R.S. (2002). Obesity and the risk of heart failure. N. Engl. J. Med..

[5] Couillard C., Ruel G., Archer W.R., Pomerleau S., Bergeron J., Couture P., Lamarche B., Bergeron N. (2005). Circulating levels of oxidative stress markers and endothelial adhesion molecules in men with abdominal obesity. J. Clin. Endocrinol. Metab..

[6] Rocha V.Z., Libby P. (2009). Obesity, inflammation, and atherosclerosis. Nat. Rev. Cardiol..

[7] Grundy S.M. (2016). Metabolic syndrome update. Trends Cardiovasc. Med..

[8] Engin A. (2017). Endothelial dysfunction in obesity. Adv. Exp. Med. Biol..

[9] (2021). WHO Obesity and Overweight Fact Sheet. https://www.who.int/news-room/fact-sheets/detail/obesity-and-overweight.

[10] Congdon P., Amugsi D. (2022). Editorial: The obesity epidemic: Causes, context, prevention. Front. Public Health.

[11] Fried S.K., Rao S.P. (2003). Sugars, hypertriglyceridemia, and cardiovascular disease. Am. J. Clin. Nutr..

[12] Mensink R.P., Zock P.L., Kester A.D., Katan M.B. (2003). Effects of dietary fatty acids and carbohydrates on the ratio of serum total to HDL cholesterol and on serum lipids and apolipoproteins: A meta-analysis of 60 controlled trials. Am. J. Clin. Nutr..

[13] Welsh J.A., Sharma A., Abramson J.L., Vaccarino V., Gillespie C., Vos M.B. (2010). Caloric sweetener consumption and dyslipidaemia among US adults. J. Am. Med. Assoc..

[14] Aeberli I., Gerber P.A., Hochuli M., Kohler S., Haile S.R., Gouni-Berthold I., Berthold H.K., Spinas G.A., Berneis K. (2011). Low to moderate sugar-sweetened beverage consumption impairs glucose and lipid metabolism and promotes inflammation in healthy young men: A randomized controlled trial. Am. J. Clin. Nutr..

[15] Brown I.J., Stamler J., Van Horn L., Robertson C.E., Chan Q., Dyer A.R., Huang C.C., Rodriguez B.L., Zhao L., Daviglus M.L. (2011). Sugar-sweetened beverage, sugar intake of individuals, and their blood pressure: International study of macro/micronutrients and blood pressure. Hypertension.

[16] Iqbal M.P. (2014). Trans fatty acids—A risk factor for cardiovascular disease. Pak. J. Med. Sci..

[17] Grillo A., Salvi L., Coruzzi P., Salvi P., Parati G. (2019). Sodium intake and hypertension. Nutrients.

[18] Daniel N., Rossi Perazza L., Varin T.V., Trottier J., Marcotte B., St-Pierre P., Barbier O., Chassaing B., Marette A. (2021). Dietary fat and low fiber in purified diets differently impact the gut-liver axis to promote obesity-linked metabolic impairments. Am. J. Physiol. Gastrointest. Liver Physiol..

[19] Harris W.S., Tintle N.L., Imamura F., Qian F., Korat A.V.A., Marklund M., Djoussé L., Bassett J.K., Carmichael P.H., Chen Y.Y. (2021). Blood n-3 fatty acid levels and total and cause-specific mortality from 17 prospective studies. Nat. Commun..

[20] Jiang L., Wang J., Xiong K., Xu L., Zhang B., Ma A. (2021). Intake of Fish and Marine n-3 Polyunsaturated Fatty Acids and Risk of Cardiovascular Disease Mortality: A Meta-Analysis of Prospective Cohort Studies. Nutrients.

[21] Dontas A.S., Zerefos N.S., Panagiotakos D.B., Vlachou C., Valis D.A. (2007). Mediterranean diet and prevention of coronary heart disease in the elderly. Clin. Interv. Aging.

[22] Hu E.A., Steffen L.M., Coresh J., Appel L.J., Rebholz C.M. (2020). Adherence to the Healthy Eating Index-2015 and Other Dietary Patterns May Reduce Risk of Cardiovascular Disease, Cardiovascular Mortality, and All-Cause Mortality. J. Nutr..

[23] Teicholz N. (2023). A short history of saturated fat: The making and unmaking of a scientific consensus. Curr. Opin. Endocrinol. Diabetes Obes..

[24] EFSA Panel on Dietetic Products, Nutrition, and Allergies (NDA) (2010). Scientific Opinion on Dietary Reference Values for fats, including saturated fatty acids, polyunsaturated fatty acids, monounsaturated fatty acids, trans fatty acids, and cholesterol. EFSA J..

[25] Ravaut G., Légiot A., Bergeron K.F., Mounier C. (2020). Monounsaturated Fatty Acids in Obesity-Related Inflammation. Int. J. Mol. Sci..

[26] Cao X., Xia J., Zhou Y., Wang Y., Xia H., Wang S., Liao W., Sun G. (2022). The Effect of MUFA-Rich Food on Lipid Profile: A Meta-Analysis of Randomized and Controlled-Feeding Trials. Foods.

[27] Georgiadi A., Kersten S. (2012). Mechanisms of gene regulation by fatty acids. Adv. Nutr..

[28] Berland C., Cansell C., Hnasko T.S., Magnan C., Luquet S. (2016). Dietary triglycerides as signaling molecules that influence reward and motivation. Curr. Opin. Behav. Sci..

[29] de Carvalho C.C.C.R., Caramujo M.J. (2018). The Various Roles of Fatty Acids. Molecules.

[30] Kris-Etherton P.M., Harris W.S., Appel L.J., American Heart Association (2002). Nutrition Committee. Fish consumption, fish oil, omega-3 fatty acids, and cardiovascular disease. Circulation.

[31] Skulas-Ray A.C., Wilson P.W.F., Harris W.S., Brinton E.A., Kris-Etherton P.M., Richter C.K., Jacobson T.A., Engler M.B., Miller M., Robinson J.G. (2019). Omega-3 Fatty Acids for the Management of Hypertriglyceridemia: A Science Advisory from the American Heart Association. Circulation.

[32] Santos H.O., Price J.C., Bueno A.A. (2020). Beyond Fish Oil Supplementation: The Effects of Alternative Plant Sources of Omega-3 Polyunsaturated Fatty Acids upon Lipid Indexes and Cardiometabolic Biomarkers-An Overview. Nutrients.

[33] Santos H.O., May T.L., Bueno A.A. (2023). Eating more sardines instead of fish oil supplementation: Beyond omega-3 polyunsaturated fatty acids, a matrix of nutrients with cardiovascular benefits. Front. Nutr..

[34] Chatterjee A., Sharma A., Chen M., Toy R., Mottola G., Conte M.S. (2014). The pro-resolving lipid mediator maresin 1 (MaR1) attenuates inflammatory signaling pathways in vascular smooth muscle and endothelial cells. PLoS ONE.

[35] Peña-de-la-Sancha P., Muñoz-García A., Espínola-Zavaleta N., Bautista-Pérez R., Mejía A.M., Luna-Luna M., López-Olmos V., Rodríguez-Pérez J.M., Fragoso J.M., Carreón-Torres E. (2023). Eicosapentaenoic and Docosahexaenoic Acid Supplementation Increases HDL Content in n-3 Fatty Acids and Improves Endothelial Function in Hypertriglyceridemic Patients. Int. J. Mol. Sci..

[36] Moreno C., Macías A., Prieto A., De la Cruz A., González T., Valenzuela C. (2012). Effects of n-3 polyunsaturated fatty acids on cardiac ion channels. Front. Physiol..

[37] Billman G.E., Harris W.S. (2011). Effect of dietary omega-3 fatty acids on the heart rate and the heart rate variability responses to myocardial ischemia or submaximal exercise. Am. J. Physiol. Heart Circ. Physiol..

[38] Christensen J.H., Christensen M.S., Dyerberg J., Schmidt E.B. (1999). Heart rate variability and fatty acid content of blood cell membranes: A dose-response study with n-3 fatty acids. Am. J. Clin. Nutr..

[39] O’Keefe J.H., Abuissa H., Sastre A., Steinhaus D.M., Harris W.S. (2006). Effects of omega-3 fatty acids on resting heart rate, heart rate recovery after exercise, and heart rate variability in men with healed myocardial infarctions and depressed ejection fractions. Am. J. Cardiol..

[40] WHO (1995). Physical Status: The Use and Interpretation of Anthropometry: Report of a WHO Expert Committee.

[41] Boldarine V.T., Joyce E., Pedroso A.P., Telles M.M., Oyama L.M., Bueno A.A., Ribeiro E.B. (2021). Oestrogen replacement fails to fully revert ovariectomy-induced changes in adipose tissue monoglycerides, diglycerides and cholesteryl esters of rats fed a lard-enriched diet. Sci. Rep..

[42] Wilson F.N., Macleod A., Barker P.S. (1931). The potential variations produced by the heart beat at the apices of Einthoven’s triangle. Am. Heart J..

[43] R Core Team (2022). R: A Language and Environment for Statistical Computing.

[44] Tibbe T.D., Montoya A.K. (2022). Correcting the bias correction for the bootstrap confidence interval in mediation analysis. Front. Psychol..

[45] WHO (2008). Waist Circumference and Waist-Hip Ratio: Report of a WHO Expert Consultation.

[46] Breheny P., Burchett W. (2017). Visualization of regression models using visreg. R J..

[47] Rautaharju P.M., Zhang Z.M., Gregg R.E., Haisty W.K., ZVitolins M., Curtis A.B., Warren J., Horaĉek M.B., Zhou S.H., Soliman E.Z. (2013). Normal standards for computer-ECG programs for prognostically and diagnostically important ECG variables derived from a large ethnically diverse female cohort: The Women’s Health Initiative (WHI). J. Electrocardiol..

[48] Meek S., Morris F. (2002). Introduction. II—Basic terminology. BMJ.

[49] Vepsäläinen T., Laakso M., Lehto S., Juutilainen A., Airaksinen J., Rönnemaa T. (2014). Prolonged P wave duration predicts stroke mortality among type 2 diabetic patients with prevalent non-major macrovascular disease. BMC Cardiovasc. Disord..

[50] Rijnbeek P.R., van Herpen G., Bots M.L., Man S., Verweij N., Hofman A., Hillege H., Numans M.E., Swenne C.A., Witteman J.C. (2014). Normal values of the electrocardiogram for ages 16–90 years. J. Electrocardiol..

[51] Bennett D.H., Evans D.W. (1974). Correlation of left ventricular mass determined by echocardiography with vectorcardiographic and electrocardiographic voltage measurements. Br. Heart J..

[52] Garcia-Pavia P., Rapezzi C., Adler Y., Arad M., Basso C., Brucato A., Burazor I., Caforio AL P., Damy T., Eriksson U. (2021). Diagnosis and treatment of cardiac amyloidosis: A position statement of the ESC Working Group on Myocardial and Pericardial Diseases. Eur. Heart J..

[53] Gersovitz M., Madden J.P., Smiciklas-Wright H. (1978). Validity of the 24-hr. dietary recall and seven-day record for group comparisons. J. Am. Diet. Assoc..

[54] Simopoulos A.P. (1991). Omega-3 fatty acids in health and disease and in growth and development. Am. J. Clin. Nutr..

[55] Sheppard K.W., Cheatham C.L. (2018). Omega-6/omega-3 fatty acid intake of children and older adults in the U.S.: Dietary intake in comparison to current dietary recommendations and the Healthy Eating Index. Lipids Health Dis..

[56] Marangoni F., Colombo C., Martiello A., Negri E., Galli C. (2007). The fatty acid profiles in a drop of blood from a fingertip correlate with physiological, dietary and lifestyle parameters in volunteers. Prostaglandins Leukot. Essent. Fat. Acids.

[57] Min Y., Ghebremeskel K., Geppert J., Khalil F. (2011). Effect of storage temperature and length on fatty acid composition of fingertip blood collected on filter paper. Prostaglandins Leukot. Essent. Fat. Acids.

[58] Meyer B.J., Sparkes C., Sinclair A.J., Gibson R.A., Else P.L. (2021). Fingertip Whole Blood as an Indicator of Omega-3 Long-Chain Polyunsaturated Fatty Acid Changes during Dose-Response Supplementation in Women: Comparison with Plasma and Erythrocyte Fatty Acids. Nutrients.

[59] Makrides M., Neumann M.A., Byard R.W., Simmer K., Gibson R.A. (1994). Fatty acid composition of brain, retina, and erythrocytes in breast- and formula-fed infants. Am. J. Clin. Nutr..

[60] Komatsuzaki N., Eda A., Kameoka R., Nakashima Y. (2013). Effects of Intake of Maternal Dietary Elaidic Acids during Pregnancy and Lactation on the Fatty Acid Composition of Plasma, Erythrocyte Membrane, and Brain in Rat Pups. J. Nutr. Metab..

[61] Nikolaidis M.G., Petridou A., Mougios V. (2006). Comparison of the phospholipid and triacylglycerol fatty acid profile of rat serum, skeletal muscle and heart. Physiol. Res..

[62] Nodera M., Suzuki H., Yamada S., Kamioka M., Kaneshiro T., Kamiyama Y., Takeishi Y. (2015). Association of Serum n-3/n-6 Polyunsaturated Fatty Acid Ratio with T-Wave Alternans in Patients with Ischemic Heart Disease. Int. Heart J..

[63] McLennan P.L. (2014). Cardiac physiology and clinical efficacy of dietary fish oil clarified through cellular mechanisms of omega-3 polyunsaturated fatty acids. Eur. J. Appl. Physiol..

[64] Ramadeen A., Connelly K.A., Leong-Poi H., Hu X., Fujii H., Laurent G., Domenichiello A.F., Bazinet R.P., Dorian P. (2012). Docosahexaenoic Acid, but Not Eicosapentaenoic Acid, Supplementation Reduces Vulnerability to Atrial Fibrillation. Circ. Arrhythm. Electrophysiol..

[65] Innes J.K., Calder P.C. (2018). The Differential Effects of Eicosapentaenoic Acid and Docosahexaenoic Acid on Cardiometabolic Risk Factors: A Systematic Review. Int. J. Mol. Sci..

[66] Sherrard G. (2023). Are Anthropometric Biomarkers, Nutrient Intake and Blood Fatty Acid Composition Associated with the Electrical Activity of the Heart in a Sample Population of Healthy Women?. Master’s Thesis.

