# Supplementary material for: Capillary Blood Docosahexaenoic Acid Levels Predict Electrocardiographic Markers in a Sample Population of Premenopausal Women
Source: J Clin Med. 2024 Oct 7;13(19):5957. doi: 10.3390/jcm13195957 (PMC11478101; doi:10.3390/jcm13195957)
Supplement: Supplementary file 1 [file jcm-13-05957-s001.zip › jcm-3203846-supplementary.pdf]

Supplementary data for:

Capillary Blood Docosahexaenoic Acid Levels Predict Electrocardiographic Markers in a Sample Population of Premenopausal Women

Breno P. Casagrande [1,2,†], George Sherrard [1,†], Mike S. Fowler [3], Débora Estadella [2] and Allain A. Bueno [1,*]

1 College of Health, Life and Environmental Sciences, University of Worcester, Henwick Grove, Worcester WR2 6AJ, UK; breno.casagrande@unifesp.br (B.P.C.); georgiehome@yahoo.co.uk (G.S.)

2 Biosciences Department, Institute of Health and Society, Federal University of S.o Paulo, Santos 1015-020, SP, Brazil; estadella@unifesp.br

3 Department of Biosciences, Swansea University, Singleton Park, Swansea SA2 8PP, UK; m.s.fowler@swansea.ac.uk

* Correspondence: a.bueno@worc.ac.uk; Tel.: +44-01905-542525

† These authors contributed equally to this work.

Supplementary Table S1. Full dietary analysis, 4-day average consumption for each nutritional component.

Nutritional component / daily consumption	Mean ± SD or Median (IQR)
Biotin (B7) (ug) #	29.067 (IQR 15.51, 38.402)
Calcium (mg)	509.079 (SD 202.104)
Carbohydrate (g)	164.355 (SD 65.177)
Carbohydrates (% total energy)	42.03 (SD: 9.86)
Carotene (ug) #	2027.803 (IQR 627.253, 3588.422)
Chloride (mg)	2673.424 (SD 945.769)
Cholesterol (mg)	199.15 (SD: 125.48)
Copper (mg)	0.809 (SD 0.37)
Energy (Total Kcal)	1577.783 (SD 408.693)
Fibre (g)	17.99 (SD: 6.00)
Folates (B9) Total (ug) #	200.648 (IQR 135.658, 259.445)
Free Sugars (g) #	14.88 (IQR 7.69, 28.36)
Fructose (g) #	8.19 (IQR 3.502, 12.058)
Galactose (g) #	0.002 (IQR 0.001, 0.048)
Glucose (g)	7.173 (SD 4.773)
Iodine (ug)	82.668 (SD 44.905)
Iron (mg) #	8.538 (IQR 5.516, 10.241)
Lactose (g)	6.517 (SD 4.155)
Magnesium (mg)	195.849 (SD 65.498)
Maltose (g) #	0.746 (IQR 0.471, 1.554)
Manganese (mg) #	1.735 (IQR 1.428, 2.746)
MUFA (% total energy)	9.58 (SD: 4.38)
MUFA (g)	15.61 (SD: 7.13)
N-3 Total (g)	0.91 (SD: 0.82)
N-6 Total (g) #	2.82 (IQR 2.04, 3.97)
N-6:N-3 Ratio	5.55 (SD: 4.57)
Niacin (mg)	15.156 (SD 6.318)
Niacin total (B3) (mg)	25.128 (SD 11.845)
NSP (g)	13.556 (SD 5.007)
Oligosaccharides (g) #	0.26 (IQR 0.135, 0.61)
Pantothenic Acid (B5) (mg)	4.528 (SD 2.381)
Phosphorus (mg)	794.838 (SD 274.974)
Potassium (mg)	1982.037 (SD 700.821)
Protein (% total energy)	19.16 (SD: 4.07)
Protein (g) #	71.86 (IQR 66.86, 77.96)
PUFA (% total energy)	4.36 (SD: 2.04)
PUFA (g)	7.15 (SD: 3.54)
Retinol (ug) #	173.786 (IQR 101.171, 246.818)

Riboflavin (B2) (mg)	1.409 (SD 0.719)
Selenium (ug) #	34.723 (IQR 26.697, 42.865)
SFA (% total energy)	14.03 (SD: 4.40)
SFA (g)	23.41 (SD: 8.27)
Sodium (mg)	1762.358 (SD 586.261)
Starch (g)	93.156 (SD 36.353)
Sucrose (g) #	11.543 (IQR 5.783, 20.924)
Sugars (g)	68.237 (SD 36.126)
TFA (% total energy)	0.26 (SD: 0.13)
TFA (g) #	0.35 (IQR 0.24, 0.57)
Thiamine (B1) (mg)	1.23 (SD 0.549)
Total fat (% total energy)	38.81 (SD: 10.00)
Total fat (g)	64.54 (SD: 19.49)
Tryptophan (mg)	580.201 (SD 228.403)
Vitamin A (ret eq) (ug)	707.283 (SD 469.757)
Vitamin B12 (ug) #	3.566 (IQR 2.097, 4.331)
Vitamin B6 (mg)	1.539 (SD 0.781)
Vitamin C (mg) #	69.587 (IQR 46.234, 112.794)
Vitamin D (ug) #	3.644 (IQR 1.66, 4.65)
Vitamin E (mg) #	5.113 (IQR 3.426, 8.986)
Vitamin K 1 (ug) #	44.904 (IQR 22.746, 72.748)
Zinc (mg) #	5.681 (IQR 4.722, 6.773)

(Kcal) kilocalories; (MUFA) monounsaturated fatty acid; (PUFA) polyunsaturated fatty acid; (SFA) saturated fatty acid; (TFA) trans fatty acid; (SD) standard deviation; (IQR) interquartile range, P25 and P75; (DRV) Dietary Reference Values. # indicates non-Gaussian distribution, and the use of median and IQR for data presentation.

Supplementary Table S2. Complementary to Table 2: Peripheral blood fatty acid profile (% of fatty acids) determined by GC-FID.

**Fatty acid (% of total FAs)**	**Mean ± SD or Median (IQR)**
SFA total %	35.308 (SD 2.829)
C14:0#	0.745 (IQR 0.54, 1.008)
C16:0	23.332 (SD 2.119)
C18:0#	9.31 (IQR 7.921, 10.017)
C20:0	0.115 (SD 0.043)
C22:0	0.304 (SD 0.141)
C24:0	1.943 (SD 0.44)
MUFA total %	25.193 ± 3.000
C16:1n-7#	1.067 (IQR 0.651, 1.382)
C18:1n-9	20.483 (SD 2.374)

C18:1n-7	1.556 (SD 0.365)
C20:1n-9	0.397 (SD 0.143)
C22:1n-9	0.058 (IQR 0.051, 0.074)
C24:1n-9#	1.487 (SD 0.350)
PUFA total %	37.89 (SD 4.189)
n-6 total %	32.939 (SD 3.685)
C18:2n-6	22.692 (SD 3.556)
C18:3n-6#	0.133 (IQR 0.096, 0.143)
C20:2n-6	0.098 (SD 0.018)
C20:3n-6	1.247 (SD 0.283)
C20:4n-6	8.769 (SD 1.501)
n-3 total %	4.953 (SD 0.788)
C18:3n-3#	0.29 (IQR 0.205, 0.34)
C20:3n-3#	0.21 (IQR 0.163, 0.276)
C20:5n-3#	0.43 (IQR 0.391, 0.624)
C22:5n-3	0.968 (SD 0.198)
C22:6n-3	2.909 (SD 0.642)
n-6:n:3 ratio	6.752 (SD 0.924)
DMA 16:0	0.295 (SD 0.06)
∑DMA 18:x	1.312 (SD 0.292)

(DMA) dimethyl acetals of fatty acids; (GC-FID) gas chromatography - flame ionisation detection; (IQR) inter-quartile range, p25 and p75; (MUFA) monounsaturated fatty acid; (PUFA) polyunsaturated fatty acids; (SD) standard deviation; (SFA) saturated fatty acid. n=23.

Supplementary Table S3. Mediation model and Model information.

**Path**	**Predictor**	**Dependent**	**β (standardized effect)**	**z value**	**p value**	**Power (1-β)**
**Main model**						
a (X→M)	DHA	R wave amplitude	-0.533	-2.881	0.004*	0.821
b (M→Y)	R wave amplitude	AUC (QRS)	0.891	9.797	<0.001*	>0.999
c (X→Y)	DHA	AUC (QRS)	-0.138	-1.413	0.158	0.293
**Control variables**						
p1 (C1→X)	Factor 1	DHA	-0.454	-2.47927	0.013	0.698
p2 (C1→M)	Factor 1	R wave amplitude	-0.370	-2.01359	0.044	0.521

p3 (C1→Y)	Factor 1	AUC (QRS)	-0.003	-0.02747	0.978	0.050
p4 (C2→X)	Factor 2	DHA	0.004	0.021858	0.983	0.050
p5 (C2→M)	Factor 2	R wave amplitude	-0.301	-1.85494	0.064	0.458
p6 (C2→Y)	Factor 2	AUC (QRS)	-0.063	-0.82237	0.411	0.130
**Effects**						
Indirect (a*b)			-0.485	-3.041	0.002*	0.860
Direct (c)			-0.138	-1.413	0.158	0.293
Total (a*b+c)			-0.632	-3.474	0.001*	0.935

(X) predictor; (M) mediator; (Y) dependant; (C1 and C2) control variables. * Indicates significative correlations with power above the required value (0.8). n=23
